# Nonlinear Dynamics of a Star-Shaped Structure and Variable Configuration of Elastic Elements for Energy Harvesting Applications

**DOI:** 10.3390/s22072518

**Published:** 2022-03-25

**Authors:** Jerzy Margielewicz, Damian Gąska, Grzegorz Litak, Piotr Wolszczak, Carlo Trigona

**Affiliations:** 1 Faculty of Transport and Aviation Engineering, Silesian University of Technology, 40-019 Katowice, Poland; jerzy.margielewicz@polsl.pl (J.M.); damian.gaska@polsl.pl (D.G.); 2 Faculty of Mechanical Engineering, Lublin University of Technology, 20-618 Lublin, Poland; p.wolszczak@pollub.pl; 3 Dipartimento di Ingegneria Elettrica Elettronica ed Informatica, University of Catania, 95125 Catania, Italy; carlo.trigona@dieei.unict.it

**Keywords:** bifurcations, Lyapunov exponent, chaotic attractors, periodicity, energy efficiency

## Abstract

The subject of the model research contained in this paper is a new design solution of the energy harvesting system with a star-shaped structure of elastic elements and variable configuration. Numerical experiments focused mainly on the assessment of the configuration of elastic elements in the context of energy harvesting efficiency. The results of computer simulations were limited to zero initial conditions as it is the natural position of the static equilibrium. The article compares the energy efficiency for the selected range of the dimensionless excitation frequency. For this purpose, four cases of elastic element configurations were compared. The results are visualized based on the diagram of RMS voltage induced on piezoelectric electrodes, bifurcation diagrams, Lyapunov exponents, and Poincaré maps, showing the impact of individual solutions on the efficiency of energy harvesting. The results of the simulations show that the harvester’s efficiency ranges from 4 V to 20 V depending on the configuration and the frequency range of the excitation, but the design allows for a smooth adjustment to the given conditions.

## 1. Introduction

In most engineering solutions, mechanical vibrations induced during machine operation are an unfavorable phenomenon. Their presence reduces the reliability and life of the device, which in turn leads to economic losses, and in extreme cases to failure or destruction of the machine [1,2]. Fatigue failures are very often caused by cyclically varying loads in the low frequency range. On the other hand, vibrations of machine elements, which are often a negative factor in machine operation and their complete damping is impossible [3] or ineffective, may be positive in another aspect [4]. One such aspect is the recovery of energy from mechanical vibrations and other sources by means of specially constructed devices that convert the mechanical energy of vibrations into electrical energy. Such devices are called energy harvesters [5,6].

Piezoelectric transducers are characterized by high energy density ideal for harvesting energy of small size that can be treated as a power source for sensors that monitor the operation of devices in places where access to a constant source of energy is impossible or difficult [7]. The energy efficiency measured by the amount of energy in the output of the energy harvester is small, but sufficient to power the sensors and send the collected data [8]. Since the beginning of this century, research into the application of the piezoelectric effect in the recovery of energy from vibrations has been very intensively developed. They relate to both the construction issues of energy harvesters and materials [9,10,11].

Considering the construction of energy harvesters to recover energy from mechanical vibrations, most of them are based on a flexible cantilever beam with piezoelectric transducers glued to this beam [12,13]. When the beam is excited to vibrate, it deforms, which allows it to harvest energy. The most important thing in such a system is adequate energy efficiency in a wide range of excitation frequencies. Because, during machine operation, a wide spectrum of harmonic components is excited [14], solutions based on linear systems require many parallel connected subsystems. Each of these is tuned to a different frequency [15]. This is a major disadvantage of linear systems. Therefore, at present, nonlinear systems are being designed and studied that have a much wider spectrum of applications [16,17,18,19]. The potential barrier in such systems is mainly shaped by magnets on both the beam and the energy harvester housing. In multimodal systems, widely developed in recent years, nonlinearity is also introduced by e.g., considering large displacements of the beams [20,21]. Other design solutions are also known, consisting of two flexible beams, coupled by electric circuits [22] and mechanical systems [23,24]. There are also systems based on single free and coupled pendulums [25] or double pendulums [26,27]. The current scientific challenges in the field of energy harvesters are increasing their efficiency and output power, adaptation to various conditions, and miniaturization as well as work in the field of high-energy orbits. Moreover, nonlinear energy harvesters are highly sensitive to uncertainty indicators [28]. Each of these systems has certain limitations. The vibration level of the external source should enable a jump between individual local potential zones because then the trajectories recorded on the phase plane reach the greatest possible displacement amplitudes.

Alternative design solutions for energy harvesting systems are those in which the energy potential is characterized by properly connected elastic elements of the variable configuration. Their advantage is the ability to adapt to specific excitation conditions, in particular low vibration amplitudes. The quasi-zero energy harvester (QZEH) [29] is a special design solution of the star-shaped structure system proposed in this paper. The main difference is the configuration of the spring elements through which the potential barrier can be modified. In the case of the QZEH system, we are dealing with an almost flat potential well. However, in the analyzed system, we can configure the potential barrier. This assumption was adopted in this work to design a new energy harvesting system based on elastic and damping elements and to analyze its energy efficiency for various configurations.

## 2. Formulation of the Mathematical Model

**Figure 1:**
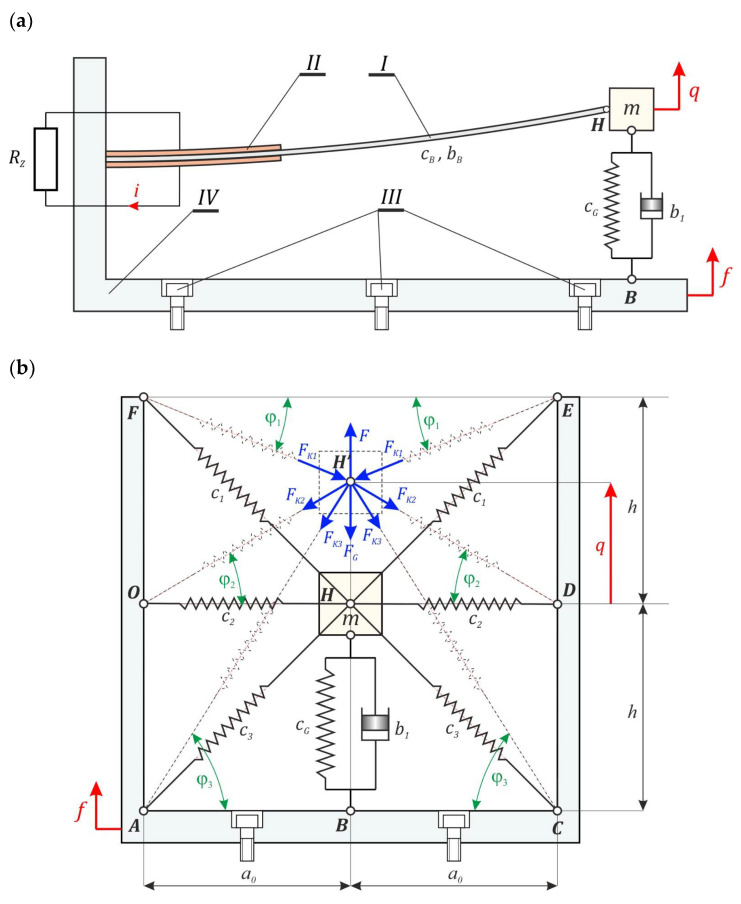
Schematic diagrams of the energy harvesting system with a star-shaped structure of elastic elements, visible from the direction: (**a**) perpendicular to the axis of the flexible beam, (**b**) parallel to the axis of the flexible beam. Geometrical and system parameters as well as the resulting force with their components are denoted in the picture.

The subject of model research is the energy harvesting system with elastic elements of variable configuration (Figure 1). A significant feature of the analyzed design solution of the energy harvesting system is the possibility of implementing them both on the macro and micro scale. The structure can be made of various materials. In the tested system, a symmetrical structure of connections of compensation springs was assumed, which are attached at points *A* and *C* to the nondeformable *IV* frame. Through bolts *III*, the system is rigidly attached to the vibrating machine element, from which energy is harvested. Piezoelectric elements *II* were glued to the flat surfaces of the flexible cantilever beam *I*, which under the influence of dynamic external influence undergoes elastic deformation. As a result of the deformation on the electrodes of the piezoelectric *II*, an electric charge is induced. Furthermore, during model tests, it was assumed that the inertial element *m*, mounted on the free end of the *I* beam, was able to move along a straight line, the direction of which was determined by the *C_G_* main spring axis. We would like to inform you here that this assumption determines the appropriate design solution of this element, which is not the subject of the considerations contained in this paper.

From the construction point of view, the considered system is an extension of the structure with quasi-zero stiffness QZEH [29]. The distinguishing elements of the design solution considered are the additional springs *c*_1_ and *c*_3_. In the tested energy harvesting system, the compensation springs form a star-shaped geometric structure, which is shown in the schematic diagram in Figure 1b. These additional linear elastic elements and the cause–effect relationships between them play a significant role in shaping the potential barrier. Moreover, during the numerical experiments, the dissipation element *b*_1_ was taken into account, which creates a parallel connection with the main spring *C_G_*. The damper *b*_1_ models the energy losses in the movable connections of the spring system with a variable configuration.

### 2.1. Identification of the Mechanical Characteristics

The formal basis for conducting numerical experiments in the field of harvesting energy is the mechanical characteristics of the transducer, which define the potential barrier. The relations between the displacement of the free end of the flexible beam *q* and the external force *F*, which loads the beam, are derived without considering the influence of inertial and dissipative forces. The external load *F*, acting on the tested energy generation system, is balanced by the forces induced in the main spring *C_G_* and the compensation springs. Furthermore, at this point, we indicate that to simplify the mathematical notation, the substitutions *y = q* − *f* and *C_Ki_ = 2C_i_* were introduced. With the above assumptions in mind, the condition of static equilibrium takes the form:

(1) $$\begin{array}{c} F=\underset{c_{Z}}{\underbrace{\left(c_{B}+c_{G}\right)}}y+c_{K1}\Delta L_{1}sin\varphi_{1}+c_{K2}\Delta L_{2}sin\varphi_{2}+c_{K3}\Delta L_{3}sin\varphi_{3}. \end{array}$$

The relations defining the lengths of the compensation springs, in the static equilibrium position *L*_0*i*_ and at the time of their deformation *L_i_*, are given with the appropriate relations: $L_{01}=\sqrt{a_{0}^{2}+h^{2}},$ $L_{1}=\sqrt{a_{0}^{2}+{\left(h-y\right)}^{2}}$, $L_{02}=\sqrt{a_{0}^{2}},$ $L_{2}=\sqrt{a_{0}^{2}+y^{2}}$*,* $L_{03}=\sqrt{a_{0}^{2}+{\left(h+y\right)}^{2}}$, and $L_{3}=\sqrt{a_{0}^{2}+{\left(h+y\right)}^{2}}$. After a few transformations, Equation (1) finally takes the form:

(2) $$\begin{array}{c} F=c_{Z}a_{0}x+2c_{K1}a_{0}\left(1-\frac{\sqrt{1+\beta^{2}}}{\sqrt{1+{\left(\beta -x\right)}^{2}}}\right)\left(\beta -x\right)+2\mu_{1}c_{K1}a_{0}x\left(1-\frac{1}{\sqrt{1+x^{2}}}\right)+\\ 2\mu_{2}c_{K1}a_{0}\left(1-\frac{\sqrt{1+\beta^{2}}}{\sqrt{1+{\left(\beta +x\right)}^{2}}}\right)\left(\beta +x\right). \end{array}$$

In the equation, there are variables which are given by relationships:$\;x=\frac{q}{a_{0}}$ and $\beta =\frac{h}{a_{0}}$. In addition, when conducting numerical experiments, the cause-and-effect relationships between the compensation springs were taken into account: $\mu_{1}=\frac{c_{K2}}{c_{K1}},\;\mu_{2}=\frac{c_{K3}}{c_{K1}},\;\mu_{3}=\frac{c_{Z}}{c_{K1}}$. Based on the mechanical characteristics of the system of springs with a variable configuration, a potential barrier is defined, which is obtained as the result of integrating relationship (2) over the generalized coordinate *x*:

(3) $$\begin{array}{c} \begin{array}{c} V=\int Fdx=\frac{1}{2}a_{0}\left(\begin{array}{c} c_{Z}x^{2}+2c_{K1}\sqrt{1+\beta^{2}}\sqrt{1+{\left(x-\beta \right)}^{2}}+2c_{K1}\beta^{2}\left(\mu_{2}-1\right)\\ -2\mu_{2}c_{K1}\sqrt{1+\beta^{2}}\sqrt{1+{\left(\beta +x\right)}^{2}}+2c_{K1}\beta x\left(1+\mu_{2}\right)\\ +c_{K1}\left(1+x^{2}\right)\left(\mu_{1}+\mu_{2}-1\right)-2\mu_{1}c_{K1}\sqrt{1+x^{2}} \end{array}\right) \end{array} \end{array}.$$

**Figure 2:**
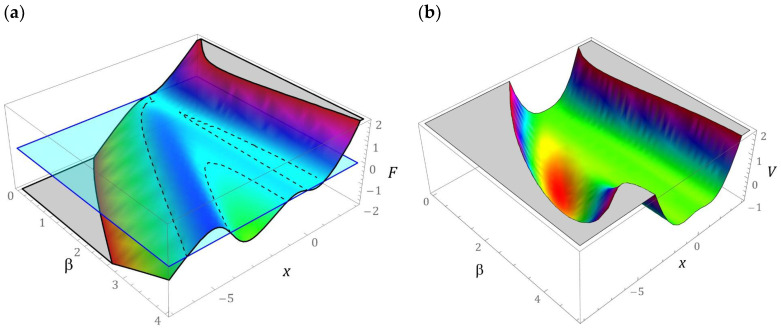
Three-dimensional images: (**a**) mechanical characteristics (restore force, *F*), (**b**) potential barrier, *V* versus tip mass displacement, *x*, and the geometrical parameter, *β*.

The three-dimensional graphs showing the evolution of the mechanical characteristics and the potential barrier due to the configuration of the compensation springs are presented in Figure 2.

**Figure 3:**
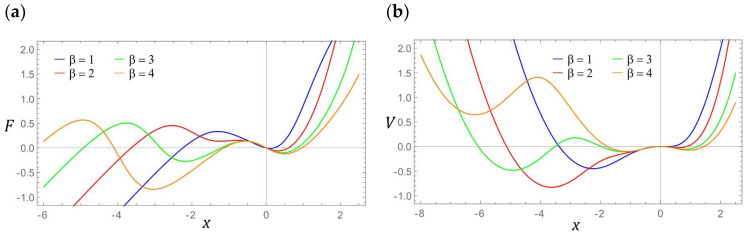
Two-dimensional images: (**a**) mechanical characteristics, *F*(*x*), (**b**) potential shape with a barrier, *V*(*x*) for various geometrical factors, *β*.

To emphasize the details related to the sensitivity of the *β* parameter, on the shape of the mechanical characteristic and the potential barrier, two-dimensional graphs have been added (Figure 3). In the special case, when *β* = 0, the system studied with a star structure of springs is reduced to the energy harvesting system with a quasi-zero potential barrier. The construction parameter *β* plays a significant role in the shape of the potential barrier. If it takes the values of *β* < 1, then we are dealing with a single well of the potential barrier. Along with its increase, the number of wells increases, and additional wells have a different depth. Regarding large values of *β* > 4, we are dealing with an energy harvesting system dominated by a potential well composed of two additional local minima separated by a relatively low energy barrier. Regardless of the value of *β*, there is an asymmetric potential characteristic. The exemplary mechanical characteristics presented in the graphs (Figure 3) will be subjected to further detailed model tests. The dependencies presented constitute a formal basis for deriving a dimensionless mathematical model based on which quantitative and qualitative numerical experiments will be carried out.

### 2.2. Mathematical Model of the Energy Harvesting System

From a mathematical point of view, the energy dissipation elements *b_B_* and *b*_1_ form a parallel connection. For this reason, a simplification was made, which comes down to the derivation of the substitute dissipation element *b_Z_*, the value of which is equal to the sum of the damping coefficients of the flexible beam *b_B_* and the energy losses in the connections of the movable compensation springs *b*_1_. The situation is similar with respect to elastic elements *C_B_* and *C_G_*. Moreover, we assume that the system is influenced by the harmonic excitation $f=Asin\left(\omega_{W}t\right)$. Bearing in mind the adopted model and simplifying assumptions, the differential equations of motion, in which the new variable *y = q* − *f* was considered, take the form:

(4) $$\left\{\begin{array}{l} m\frac{d^{2}y}{dt^{2}}+\left(b_{B}+b_{1}\right)\frac{dy}{dt}+\left(c_{B}+c_{G}\right)y+2c_{K1}\left(h-y\right)\left(1-\frac{\sqrt{a_{0}^{2}+h^{2}}}{\sqrt{a_{0}^{2}+{\left(h-y\right)}^{2}}}\right)+\\ 2c_{K2}y\left(1-\frac{\sqrt{a_{0}^{2}}}{\sqrt{a_{0}^{2}+t^{2}}}\right)+2c_{K3}\left(h+y\right)\left(1-\frac{\sqrt{a_{0}^{2}+h^{2}}}{\sqrt{a_{0}^{2}+{\left(h+y\right)}^{2}}}\right)+k_{P}u=0,\\ \frac{du}{dt}+\frac{1}{C_{P}R_{Z}}u-\frac{k_{P}}{C_{P}}\frac{dy}{dt}=0, \end{array}\right.$$

The electric system coefficients appearing in the mathematical model (6) represent, respectively, constant *k_P_*, piezoelectric capacity *C_P_*. The *R_Z_* parameter represents the equivalent resistance of the load system and the piezoelectric electric circuit. Considering the model and simplifying assumptions, the differential equations of motion in which the dimensionless displacement was taken into account ultimately take the form:

(5) $$\begin{array}{c} \left\{\begin{array}{l} \ddot{x}+\delta \dot{x}+\mu_{3}x+2\left(\beta -x\right)\left(1-\frac{\sqrt{1+\beta^{2}}}{\sqrt{1+{\left(\beta -x\right)}^{2}}}\right)+2\mu_{1}x\left(1-\frac{1}{\sqrt{1+x^{2}}}\right)+\\ 2\mu_{2}\left(\beta +x\right)\left(1-\frac{\sqrt{1+\beta^{2}}}{\sqrt{1+{\left(\beta +x\right)}^{2}}}\right)+\theta u=\omega^{2}psin\left(\omega \tau \right),\\ \dot{u}+\sigma u-\vartheta \dot{x}=0, \end{array}\right.\\ \mathrm{where:}\\ \begin{array}{c} \begin{array}{ccccccc} \mu_{1}=\frac{c_{K2}}{c_{K1}}, & \mu_{2}=\frac{c_{K3}}{c_{K1}}, & \mu_{3}=\frac{c_{B}+c_{G}}{c_{K1}}, & \omega_{0}^{2}=\frac{c_{K1}}{m}, & \delta =\frac{b_{B}+b_{1}}{m\omega_{0}}, & x=\frac{y}{a_{0}}, & \beta =\frac{h}{a_{0}}, \end{array}\\ \begin{array}{cccccc} p=\frac{A}{a_{0}}, & \theta =\frac{k_{P}}{a_{0}m\omega_{0}^{2}}, & \vartheta =\frac{k_{P}a_{0}}{C_{P}}, & \sigma =\frac{1}{\omega_{0}C_{P}R_{Z}}, & \omega =\frac{\omega_{W}}{\omega_{0}}, & \tau =\omega_{0}t. \end{array} \end{array} \end{array}$$

Based on such a formulated mathematical model of the energy harvesting system, the results of the model tests are presented in the following part of the paper.

## 3. Results

Numerical experiments mapping the dynamics of the tested energy harvesting system were carried out with reference to the numerical data summarized in Table 1.

**Figure 4:**
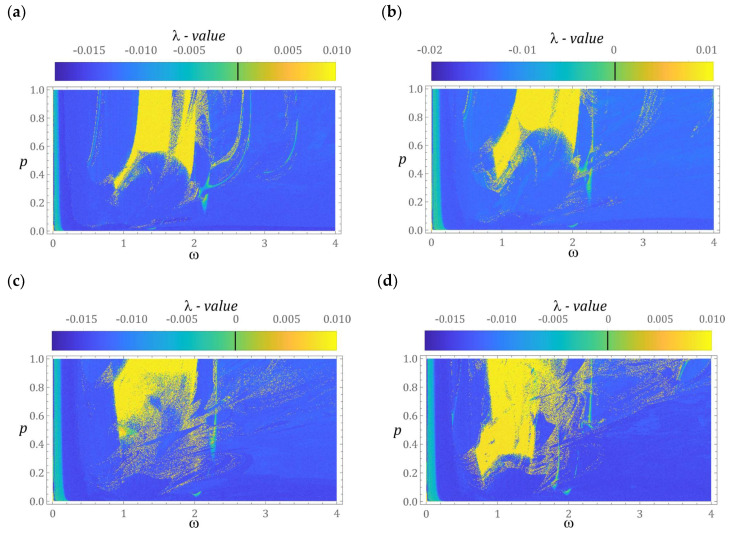
Influence of *β* on the distribution of the largest Lyapunov exponent, *λ*, for various geometrical factors, *β*: (**a**) *β* = 1, (**b**) *β* = 2, (**c**) *β* = 3, and (**d**) *β* = 4.

The first stage of the numerical experiments was to determine the distribution zones of periodic and chaotic solutions. For this purpose, a numerical procedure was used to identify the value of the largest Lyapunov exponent *λ* [30,31]. In our case, the results of computer simulations were presented in the form of two-dimensional multi-colored maps, which were plotted at a resolution of 500 × 500 points of the variability range of the control parameters *ω* and *p*. The exemplary results of computer simulations are presented in graphs in Figure 4. Multi-colored maps of the distribution of the largest Lyapunov exponent were plotted with respect to zero initial conditions. On the other hand, the values of λ in the examined area of variability of the control parameters were estimated in a three-dimensional phase space $\left(x,\;\dot{x},\;u\right)$.

The areas of chaotic solutions are distinguished by the following colors: yellow and orange (*λ* > 0). On the other hand, the shades of blue were assigned to the periodic responses (*λ* < 0). In the vicinity of *λ* ≈ 0 (green), we deal with the so-called bifurcation points. The obtained results of numerical experiments clearly indicate the dominance of periodic solutions. A direct comparison of the multi-colored maps of the distribution of the largest Lyapunov exponent (Figure 4a,b) suggests that the increase of the β-value in the examples mentioned may be due to the stretching of the multi-colored map with respect to the axis representing the dimensionless excitation amplitude.

However, in relation to the cases depicted in the graphs (Figure 4c,d), a completely opposite mechanism is observed, i.e., the multi-colored map presented in Figure 4d is the effect of compression against the axis represented by the dimensionless excitation amplitude. It is also worth noting that along with the increase in the value of the *β* parameter, the zones of unpredictable solutions expand. Most of the homogeneous area of chaotic solutions is located in the frequency band *ω* ∈ [1,2]. It is also worth noting that an increase in the value of the *β* parameter causes a shift of chaotic solutions toward low values of the dimensionless excitation amplitude. Outside this zone, point areas of solutions characterized by an unpredictable solution are excited. This situation is very clearly seen in the graphs (Figure 4c,d). Between the areas of periodic and chaotic solutions, there are basically no zones where *λ* assumes values close to 0. This situation should be explained by the large damping that occurs in the system, which is determined by the resistance to motion in the articulated joints of the elastic elements.

In the following, additional computer simulations were performed for each case of the configuration of elastic elements, which is plotted in Figure 3. The results of the numerical experiments were also carried out in relation to the zero initial conditions. This assumption of the model was made for this reason because it is the natural location of the energy harvesting system that, at the moment *t* = 0, begins to be influenced by an external dynamic excitation. As part of additional detailed model research, a direct comparison of bifurcation diagrams with diagrams of RMS voltage values induced on the electrodes was made.

It is worth mentioning here that bifurcation diagrams can be generated in several ways. One of them comes down to plotting the Poincaré cross-section points. This approach provides reliable information on the periodicity of the system’s responses. Nevertheless, the time that should be spent on drawing a bifurcation diagram with this method is much longer than the diagrams generated on the basis of local minima and maxima, or phase trajectories. In this paper, all bifurcation diagrams presented have been drawn based on local minima and maxima. This decision was made because the diagram contains information about the maximum displacements of the flexible cantilever beam. The published results clearly show that the voltage values induced on the piezoelectric electrodes directly correlate with the displacement of the beam on which the piezoelectric is attached.

In addition, the influence of the dimensionless excitation frequency on the response of the energy harvesting system was investigated. Similar studies have been carried out with respect to unpredictable solutions. The effect of this approach was to determine the influence of the configuration of elastic elements *β* and the frequency of excitation *ω* on the geometric structure of the Poincaré cross-section. The correlation dimension *D_C_* was adopted as an indicator characterizing the Poincaré cross-section. Considering the possibility of referring the results of numerical experiments to each other, computer simulations were performed for the same value of the dimensionless excitation amplitude *p*. Adopting such a model assumption will allow determining the influence of the configuration of elastic elements *β* (geometrical factor) on the efficiency of harvesting energy.

### 3.1. Model Test Results for β = 1

**Figure 5:**
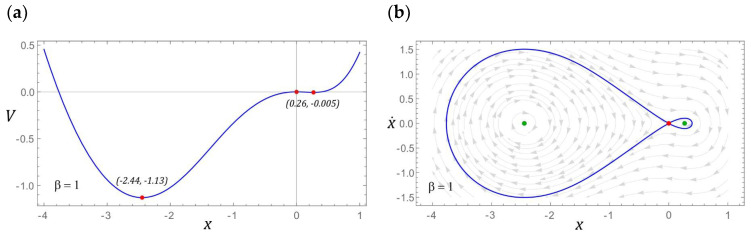
The localization of fixed points is shown as (**a**) potential characteristics, (**b**) phase plane (without damping and excitation) with the corresponding homoclinic orbits and vector field.

If the configuration of the energy harvesting system is given by the parameter *β* = 1, then the potential barrier is represented by an asymmetric function with two wells. The coordinates that characterize the position and depth of both wells are presented in Figure 5a. The depth of the well located in the positive range of the displacement values is basically minimal and amounts to *V* ≈ −0.005. The energy harvesting system, the potential barrier of which is given by this characteristic, has three fixed points, two of which are centered (green points) and a single saddle highlighted in red (Figure 5b). To illustrate the possible responses, the fixed points of the energy harvesting system were visualized against the background of the vector field and the homoclinic orbit. The representation of the vector field lines on the phase plane allows seeing how many coexisting solutions we can deal with under given load conditions of the energy harvesting system.

**Figure 6:**
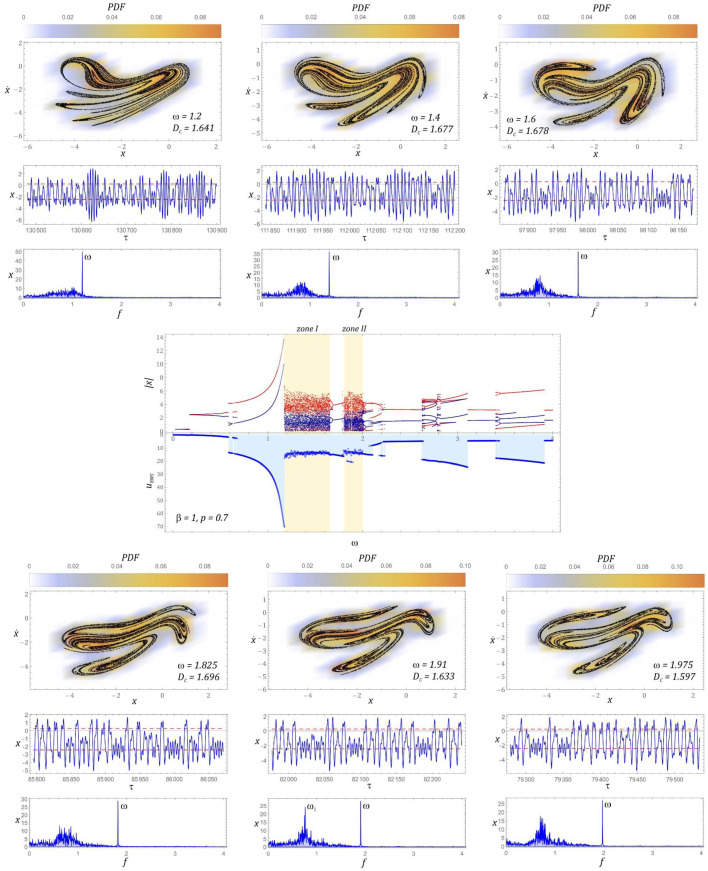
Bifurcation diagram correlated by the frequency spectra with the diagram of RMS voltage induced on piezoelectric electrodes and examples of chaotic responses (Poincaré maps with correlation dimension and probability density function (PDF) indicated).

The bifurcation diagram and the effective values of the voltage induced on the piezoelectric electrodes, for the dimensionless amplitude of mechanical vibrations *p* = 0.7, are presented in Figure 6. On the basis of computer simulations, it can be clearly stated that in the case under consideration, we are dealing with two homogeneous zones of chaotic solutions. It is worth noting that when the periodic response changes to a chaotic one, a reduction in the efficiency of energy harvesting is observed. Such a situation occurs both before the first and the second zones of unpredictable solutions. Regarding the selected values of the dimensionless excitation frequency *ω*, sample Poincaré sections illustrating their evolution were plotted. For the visualization of the cross-sections, the density of the phase trajectory intersection with the control plane was considered. Bearing in mind the quantitative characterization of the identified geometric structures, their correlation dimension *D_C_* was calculated.

In the first zone of chaotic solutions, an increase in the correlation dimension of the chaotic attractor is observed. In the second zone, the situation is the opposite, i.e., with the increase of *ω*, the correlation dimension of the geometric structure of the chaotic attractor decreases. It is worth noting that regardless of the zone of chaotic solutions, the effective value of the voltage induced on piezoelectric electrodes is set at the level of approx. *U_RMS_ ≈* 14 V. Only at the beginning of the first zone does it reach the value of 20 V and with the increase of the dimensionless excitation frequency *ω*, it decreases. In the second zone, the situation is the opposite, i.e., at the beginning of the chaotic solutions zone, the lowest effective voltage value of approx. 12 V was registered, which with the increase of *ω* increases to the level of approx. 15 V. A direct comparison of the RMS voltage values with the correlation dimensions of the Poincaré cross-section indicates that with an increase in the value of the *D_C_* correlation dimension, the efficiency of energy harvesting decreases. Regardless of the value of the dimensionless excitation frequency, almost all harmonic components located below the excitation frequency are excited in the amplitude–frequency spectra, which were drawn based on the displacement time sequences.

In the second zone, the situation is the other way around, i.e., at the beginning of the chaotic solutions zone, the lowest effective voltage value of approx. 12 V was registered, which increases to approx. 15 V. A direct comparison of the RMS voltage values with the correlation dimensions of the Poincaré cross-section of the chaotic attractor indicates that the efficiency of harvesting energy decreases with the increase of the value of the correlation dimension. Regardless of the value of the dimensionless excitation frequency, in the amplitude–frequency spectra that were plotted based on the displacement time sequences, almost all harmonic components located below the excitation frequency *ω* are excited (Figure 6).

**Figure 7:**
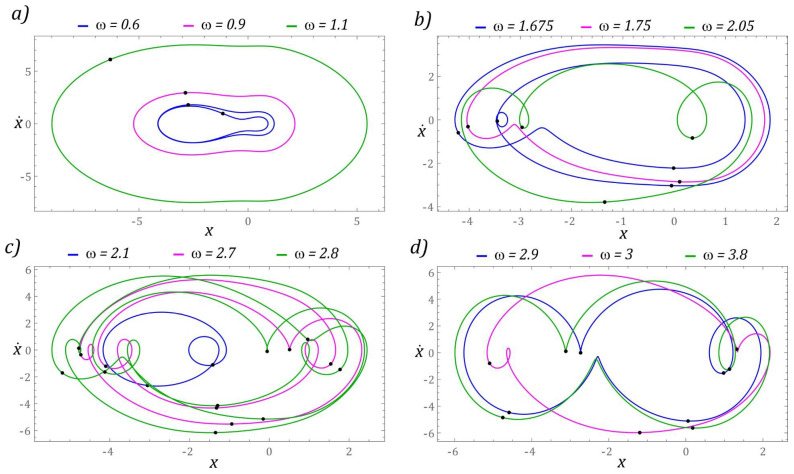
Sample graphic images of periodic solutions. The black points correspond to the Poincaré maps. The cases (**a**–**d**) show the selected solutions for increasing *ω*.

The next part presents the results of numerical experiments, characterizing periodic solutions, which are illustrated in the form of phase trajectories. Additionally, in the orbits of permanent periodic solutions, the points of intersection of the phase flow with the control plane of the Poincaré cross-section are marked (highlighted in black). The number of points directly correlates with the periodicity of the solution. The graphs in Figure 7 show the evolution of periodic solutions due to the increase in the value of *ω*.

In the range of low values *ω <* 0.6, there are periodic solutions with a periodicity of 1*T* (where *T* = 1/(2*ω*) is the period of excitation), the orbits of which are located in the potential barrier wells. At the same time, when *ω* < 0.2, the solution orbit is in the well located in the range of positive displacement values. On the other hand, the response of the system whose phase trajectory is in the well, within the range of negative displacement values, is in the range *ω* ∈ [0.2, 0.6]. This solution also occurs in the zones *ω* ∈ [2.2, 2.6], *ω* ∈ [3.1, 3.4] and for dimensionless excitation frequencies *ω* > 3.9. We do not provide graphic images of these responses as the energy harvesting capacity is negligible.

It should be noted that harvesting energy is most effective if the frequency of mechanical vibrations is in the band *ω* ∈ [1, 1.19] (Figure 7a). Graphical images of the phase trajectories of the system’s response to the excitation with the frequency of which the value is in the vicinity of the chaotic solution areas are presented in Figure 7b. In this range of *ω* variability, we deal with solutions with 2*T*, 3*T*, and 4*T* periodicity. Responses with 4*T* and 3*T* periodicity occur during the transition from a chaotic solution to a periodic one. The highest periodicities *T* > 6*T* occur in the bifurcation band *ω* ∈ [2.625, 2.8] (Figure 7c). However, for *ω* > 3, where the effective value of the voltage induced on the piezoelectric electrodes is in the range of *U_RMS_* ∈ [16, 20], the periodicity of the solutions is less than or equal to 4*T* (Figure 7d).

### 3.2. Model Test Results for β = 2

**Figure 8:**
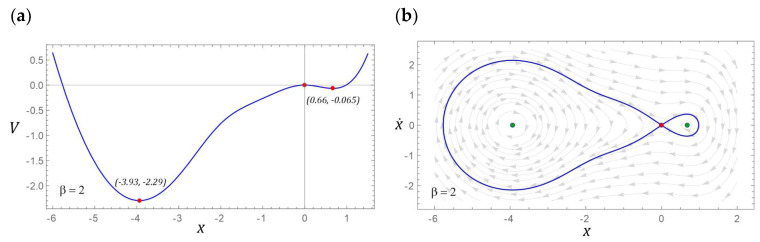
The location of fixed points is shown as (**a**) potential characteristics, (**b**) phase plane with the corresponding homoclinic orbits and vector fields.

If the *β* parameter characterizing the potential barrier takes the value 2, then there are three fixed points. Two of them represent centers and a saddle between them. Similar to the example considered in Section 3.1, the potential barrier is mapped with two wells, but this time the depths of individual wells are larger. It is also noteworthy that the homoclinic orbit resembles a deformed droplet (Figure 8b). Such an image of the homoclinic orbit is caused by the shape of one of the slopes of the well located in the range of negative displacement values (Figure 8a).

**Figure 9:**
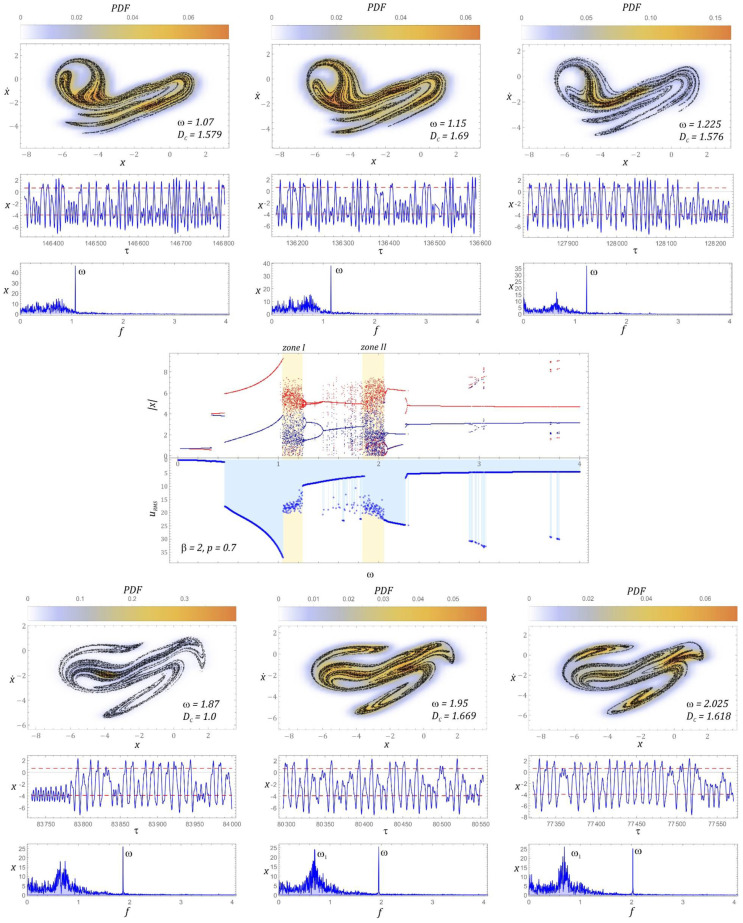
Bifurcation diagram correlated by the frequency footer with the diagram of RMS voltage induced on piezoelectric electrodes and examples of chaotic responses (Poincaré maps with correlation dimension and probability density function (PDF) indicated).

The bifurcation diagram, the diagram of the RMS values of the voltage induced on the piezoelectric electrodes, and the evolution of the chaotic attractors correlated with it are presented in Figure 9. It can be concluded that changing the value of *β* = 1 to *β* = 2 causes the areas of unpredictable solutions to shift toward lower values of the dimensionless frequency of the source of excitation. At the same time, along with the shift of the bands, a narrowing of the first zone of chaotic solutions is observed. Between homogeneous areas of unpredictable solutions, we deal in this case with the appearance of narrow bands of chaotic responses.

Contrary to the examples presented in the intersection graphs (Figure 6), the points of intersection of the phase trajectory with the control plane are not distributed relatively evenly, but in relation to the frequencies *ω =* 1.225, *ω =* 1.87 and *ω =* 2.025, the points of concentration are visible. For the case of *ω* = 1.87, we can deal with the phenomenon of transient chaos, which after a long time evolves into a periodic solution or a chaotic attractor located in the deeper potential well. This statement is justified because in fact we are dealing with a relatively small area most frequently visited by the phase trajectory.

Moreover, the amplitude–frequency spectra corresponding to the chaotic attractors, located in the range of higher values of the dimensionless excitation frequency, are characterized by much higher amplitudes of the excited harmonic components below the frequency *ω*. For the examples of *ω* = 1.87 and *ω* = 2.025, the amplitude of the excited harmonic component *ω*_1_ reaches a value comparable to the frequency amplitude *ω*. The element that significantly distinguishes the analyzed case is the almost two-fold reduction of the efficiency of obtaining energy in relation to the case of *β* = 1. Such a behavior of the system should be seen in much deeper potential wells, as a result of which, in order to achieve comparable efficiency of energy harvesting, it is necessary to influence the energy harvesting system with a signal with a much larger amplitude of vibrations. For the case of *β* = 2, the harvesting energy is most effective if *U_RMS_* reaches values larger than 25V.

**Figure 10:**
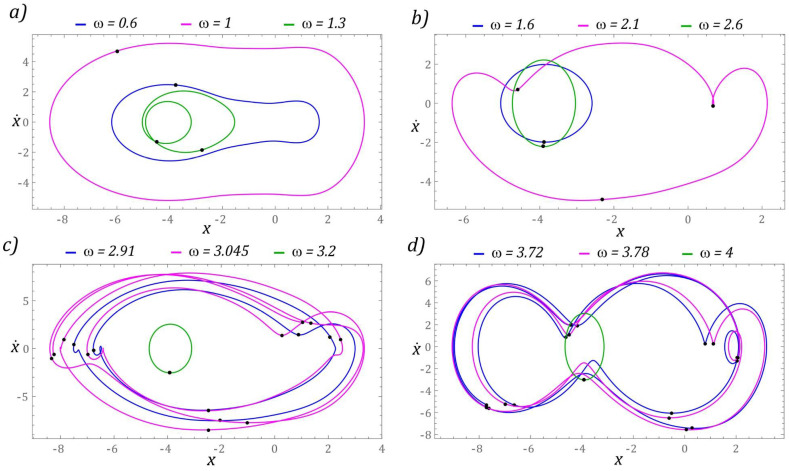
Sample graphic images of periodic solutions. The cases (**a**–**d**) show the selected solutions for increasing *ω*. The corresponding Poincaré maps with multiple black points indicate higher period solutions.

In terms of higher values of the dimensionless excitation frequency, a significant simplification of the dynamics of the energy harvesting system is observed. The wording “simplification” should be understood as limiting the periodicity of solutions. Solutions with a relatively large periodicity *T* > 6*T* occur in narrow bands located in the vicinity of *ω* = 3 and *ω ≈* 3.75. However, in the range of low values of *ω* < 0.5, similarly to the previously considered case, we are dealing with solutions located in potential wells, which are characterized by a negligible ability to harvest energy. Exemplary orbits of periodic solutions, corresponding to a given configuration *β* = 2, are presented in the graphs in Figure 10.

In the band *ω* < 1, there are periodic solutions with a periodicity of 1*T* (Figure 10a). Regardless of the value of the excitation frequency *ω* > 2.3, the response of the system is a periodic orbit with a periodicity of 1*T*, which is marked in green in the graphs (Figure 10b–d). It is worth noting that in these examples the vibration amplitude of the flexible cantilever beam is essentially unchanged. This statement is confirmed by the bifurcation diagram in Figure 9.

### 3.3. Model Test Results for β = 3

**Figure 11:**
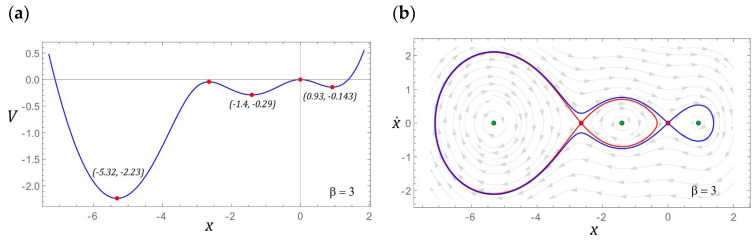
The location of fixed points is shown as (**a**) potential characteristics, (**b**) phase plane with the corresponding homoclinic orbits.

For *β* = 3, there are five fixed points in the system under study, three of which represent centers and two saddles located between them. The number of centers is directly correlated with the number of potential barrier wells. The coordinates defining the position of the potential barrier minima are given in Figure 11a. Despite the change in the value of the parameter *β*, the depth of the well located in the range of negative displacement values did not change in principle. Comparing it with the case of *β* = 2, it can be concluded that its depth slightly decreased. In the analyzed case, we deal with two homoclinic orbits, which are graphically presented in Figure 11b.

**Figure 12:**
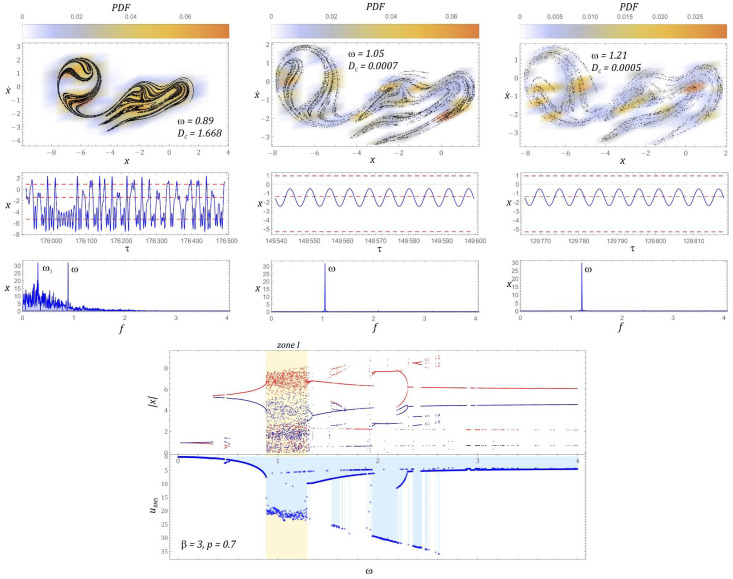
Bifurcation diagram correlated by the frequency spectra with the diagram of RMS voltage output induced on piezoelectric electrodes and examples of chaotic responses (Poincaré maps with correlation dimension and probability density function (PDF) indicated).

The graphs in Figure 12 show a bifurcation diagram and a frequency foot-coupled diagram of the RMS voltage values. With regard to the selected values of *ω*, Poincaré sections of chaotic solutions were plotted, which illustrate the evolution of attractors. For each attractor, its representations in the time and frequency domains are presented. Increasing *β* causes that in the examined case, we are dealing with a single homogeneous zone of chaotic solutions. This zone, in relation to the previously analyzed cases, has slightly shifted toward lower values of the dimensionless frequency of excitation *ω*. However, in the range of higher values *ω* > 3, the dominant solution is the solution for which the RMS voltage of *U_RMS_* ≈ 5 V is recorded on the piezoelectric electrodes.

It is worth noting that in this range of variability *ω* there are very narrow response bands, the orbits of which are located in another well of the potential barrier. If the configuration of the system is given by the parameter *β* = 3, then the best efficiency of harvesting energy at the level of *U_RMS_* ∈ [27 V, 35 V] is observed in the band *ω* ∈ [1.95, 2.6]. In other cases, the bands with the best energy harvesting efficiency were located in the vicinity of *ω* ≈ 1. Homogeneous chaotic attractors occur at the beginning of the zone of unpredictable solutions *ω* = 0.89, while in the middle zone and at the end of the zone of chaotic responses, we deal with the phenomenon of transient chaos. Chaotic attractors are attracted to a solution with a periodicity of 1*T* over time. This is confirmed by both the temporal representations of solutions as well as the plotted amplitude–frequency spectra.

**Figure 13:**
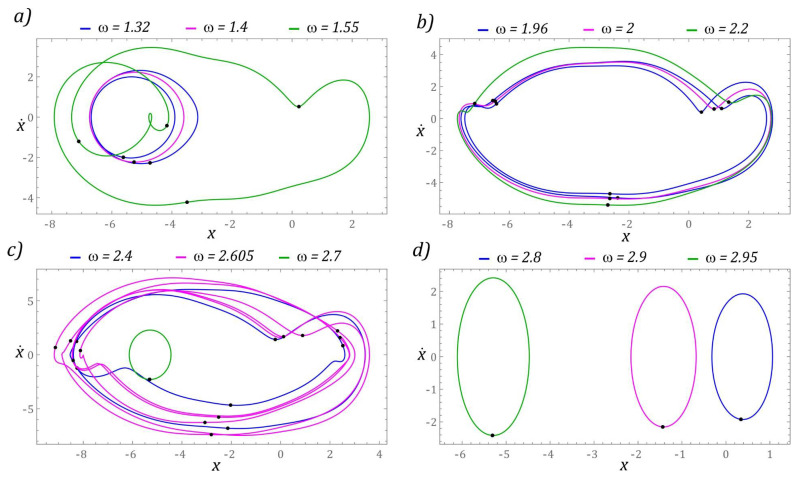
Sample graphic images of periodic solutions. The cases (**a**–**d**)show the selected solutions for increasing *ω*. The corresponding Poincaré maps with multiple black points (**a**–**c**) indicate higher period solutions.

The graphs in Figure 13 show selected periodic system responses to a given external input. Examples of solutions are depicted in the form of orbits, on the background of which are Poincaré cross-section points, representing the periodicity of solutions, are plotted.

The solutions registered just beyond the zone of chaotic solutions are characterized by a low periodicity *T* < 4*T* (Figure 13a). In the case when the dimensionless frequency of external vibrations affecting the system takes values close to *ω* = 2, a significant improvement in the efficiency of harvesting energy is observed (Figure 13b). The identified periodic solutions are characterized by orbits circling all wells of the potential barrier. The highest periodicity of the periodic solution 11*T* was recorded in the band *ω* ∈ [2.4, 2.7] (Figure 13c).

Plotting the orbits of periodic solutions made it possible to identify individual “branches” appearing in the bifurcation diagram in Figure 12. In the variability range of *ω* ∈ [2.8, 2.95], there are solutions that are located in individual wells of the potential barrier (Figure 13d). The orbits located in the outer wells of the potential barrier are located on the upper branch of which the local maxima and minima are the displacement values in the range *|x|* ∈ [4, 6]. A solution whose orbit is located in the middle well of the potential barrier occurs much less frequently. This solution in the bifurcation diagram corresponds to the branches of local minima and maxima within the range of displacement variability *|x|* ∈ [0.5, 2].

### 3.4. Model Test Results for β = 4

**Figure 14:**
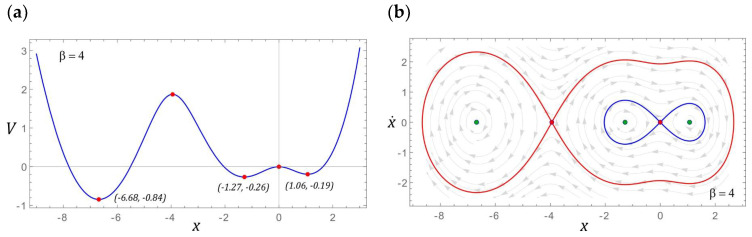
The localization of fixed points is shown as (**a**) potential characteristics, (**b**) phase plane with the corresponding homoclinic orbits and vector fields.

In the analyzed case, as in the example *β* = 3, there are three potential barrier wells (Figure 14a). Nevertheless, the distances recorded between the well minima increased. In the case under consideration, two homoclinic orbits also coexist, but their graphic images are mapped with other orbits (Figure 14b).

**Figure 15:**
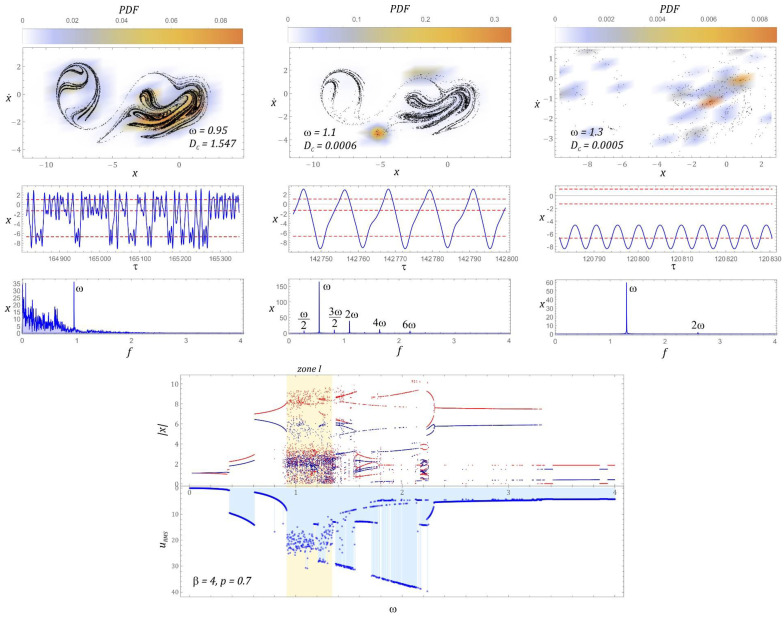
Bifurcation diagram correlated by the frequency spectra with the diagram of RMS voltage output induced on piezoelectric electrodes and examples of chaotic responses (Poincaré maps with correlation dimension and probability density function (PDF) indicated).

On the basis of the plotted characteristic, representing the shape of the potential barrier (Figure 14a), it can be concluded that the minima of individual wells are within the variability range *V* ∈ [–10]. From the point of view of the dynamics of the energy harvesting system, this property is irrelevant. A much more important parameter is the height of the local potential barrier in the vicinity of the well located in the range of negative displacement values. This amount in relation to the case of *β* = 3 increased by over 40%. Such an increase in the local potential barrier will significantly affect the efficiency of harvesting energy from vibrating mechanical devices. In fact, such a situation takes place, because in the range of low values of the dimensionless excitation frequency, the effective value of the voltage recorded on the piezoelectric electrodes reaches the maximum level of *U_RMS_*= 15V (Figure 15). For this reason, the bands showing the best ability to harvest energy *U_RMS_* ≥ 30V are in the following ranges: *ω* ∈ [1.4, 1.5] and *ω* ∈ [1.7, 2.1].

In the analyzed configuration of the energy harvesting system, there is one unpredictable solution zone, located in the band *ω* ∈ [0.9, 1.3]. Just behind the zone of chaotic responses there is a relatively wide area in which bifurcations of periodic solutions occur. It should be clearly indicated that this area is characterized by the best efficiency of harvesting energy. Regarding dimensionless excitation frequencies *ω* ≥ 2.3, there are periodic solutions with a periodicity of 1*T*, the orbits of which are located inside the potential barrier well.

Similar to the case of *β* = 3, homogeneous chaotic attractors appear at the beginning of the unpredictable response zone *ω* = 0.95 (Figure 15). In the middle and at the end of the band, we deal with the so-called phenomenon of transient chaos. In these parts of the band of unpredictable solutions, chaotic attractors are attracted to periodic orbits over time. In the middle part *ω* = 1.1, the attractive orbit goes around all wells of the potential barrier. On the other hand, at the end of the unpredictable solutions band, *ω* = 1.3, the target attractive orbit is in a well located in the range of negative displacement values. The excited harmonics in the amplitude–frequency spectra of the orbits to which the chaotic attractors are attracted are identified in the graphs in Figure 15.

In the following, examples of the orbits of permanent periodic solutions are presented, which were plotted in relation to selected values of the dimensionless excitation frequency *ω*. At the same time, for the sake of the readability of the charts, the bands in which we deal with solutions with high periodicity will be omitted. This approach is justified because a direct comparison of the bifurcation diagram with the diagram of the RMS values of the voltage induced on the piezoelectric electrodes indicates that efficient energy harvesting takes place when the response is given as a low-term solution.

**Figure 16:**
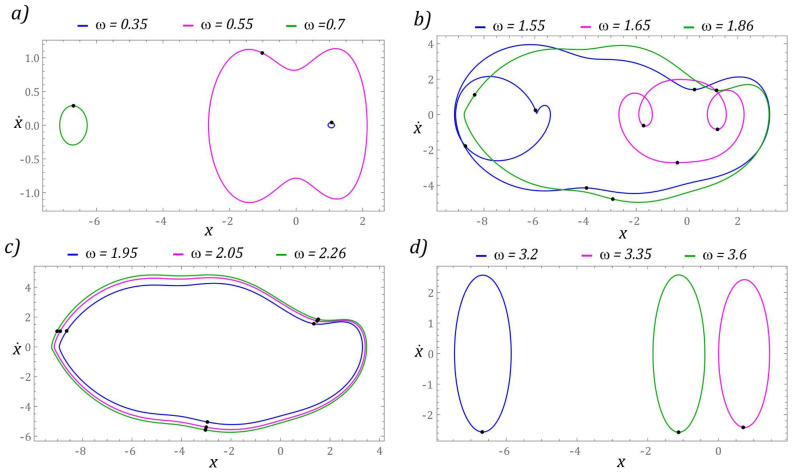
Sample graphic images of periodic solutions. The cases (**a**–**d**) show the selected solutions for increasing *ω*. The corresponding Poincaré maps with multiple black points (**b**,**c**) indicate higher period solutions.

Orbits of periodic solutions corresponding to dimensionless frequencies of the source of excitation located in the range of low values *ω* < 1 are graphically depicted in Figure 16a. In this case, 1T periodic permanent solutions are located in external wells. However, in relation to the frequency *ω* = 0.55, the orbit of the solution runs around two wells of the potential barrier. In Figure 16b, we are dealing with large orbits which oscillate around the potential barrier well. By means of these solutions, the voltage on the piezoelectric electrodes is recorded, the effective value of which reaches *U_RMS_* ∈ [30 V, 35 V]. On the other hand, between the zones characterized by the best efficiency of harvesting energy, the system’s responses are mapped by the oscillating orbit between the central well and the well located in the range of positive displacement values.

The large orbits through which the harvest of energy from vibrating mechanical systems is most efficient are shown in Figure 16c. At the same time, from the topological point of view, the included phase trajectories of the solutions included show a significant similarity. The orbit trajectories of permanent periodic solutions, located in the potential barrier wells, are presented in Figure 16b. In this case, the solutions’ direct reference to the presented solutions to the bifurcation diagram makes it possible to determine the branches of the diagram corresponding to the well in which the solution is located.

### 3.5. Comparison of the Energy Harvesting Efficiency

The published results of numerical experiments, in particular bifurcation diagrams and diagrams of effective values of the voltage induced on piezoelectric electrodes, indicate that effective energy harvesting is significantly dependent on the configuration of the elastic elements of the energy harvesting system with a star-shaped structure. For this reason, in the last part of the work, research was carried out to determine the efficiency of energy harvesting in selected ranges of variability of the dimensionless frequency of the source of excitation *ω*.

**Figure 17:**
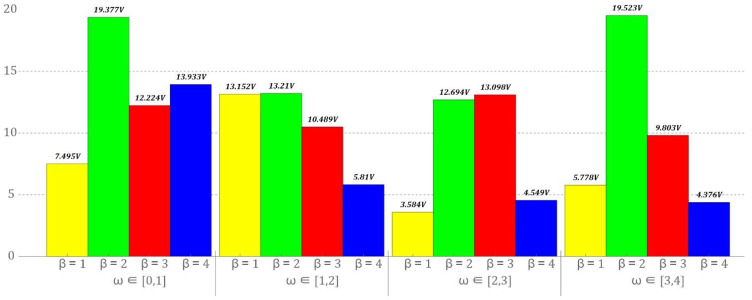
Comparison of the efficiency of harvesting energy in selected bands of variability of the dimensionless excitation frequency *ω*.

The presented analyzes are based on diagrams of the RMS values of the voltage induced on the piezoelectric electrodes. The plotted diagrams provide information on the efficiency of energy harvesting in relation to the set values *ω* bearing in mind that the dynamics of mechanical devices differ in the spectrum of excited harmonics. The decisive components in relation to energy harvesting systems are the dominant harmonics in the amplitude–frequency spectrum. For this reason, it is necessary to adjust the energy harvesting system to the given conditions. In the case of systems in which the potential barrier is shaped by the appropriate placement of permanent magnets, the tuning of the system basically comes down to designing a new energy harvesting system. In the design solution proposed in this paper, the potential barrier can be shaped in any way by appropriately changing the configuration of the elastic elements. Figure 17 shows the results of numerical calculations showing the efficiency of energy harvesting in various ranges of variability *ω*. We note that in this case, the specific voltage value is not an indicator of efficiency, because each tested configuration has different values. For this reason, we do not define a specific value as a criterion of effectiveness but only refer to the maximum value in each case, because only selected configurations were tested in the study.

The numerical data of the RMS voltage values presented in Figure 17 represent average values in the given ranges of variation *ω*. Regardless of the zone in which the vibration frequency of the mechanical device from which the energy is recovered is located, the optimal configuration of the energy harvesting system with a star-shaped structure is given by the parameter *β* = 2. The authors of the work are aware of the fact that in individual bands of variability *ω* other configurations show larger energy harvesting efficiency. Nevertheless, the existing differences are so small that, in principle, they can be ignored. The least favorable configuration of elastic elements of the tested system, *β* = 1, can be effectively used only in the band *ω* ∈ [1, 2]. The situation is similar when the configuration of elastic elements is represented by the parameter *β* = 4. With the use of such oriented elastic elements, relatively efficient energy harvesting takes place in the band *ω* ∈ [0, 1]. The configuration of elastic elements is oriented in such a way that *β* = 3 can be used effectively when the frequency of vibrations that affect the system is in the band *ω* ∈ [2, 3].

## 4. Conclusions

The paper presents a detailed study of the dynamics of a novel design solution of the energy harvesting system, with particular emphasis on the impact of its configuration on the efficiency of energy recovery. The presented results of the numerical experiments were limited to zero initial conditions. This approach is supported by the fact that at the time τ = 0 the flexible cantilever beam is in the position of static equilibrium, which in fact corresponds to zero initial conditions.

Based on the model tests of the energy harvesting system with a star-shaped structure of elastic elements with a variable configuration, it is possible to formulate the following final conclusions.

- The proposed energy harvesting system enables the potential barrier to be shaped in a relatively wide range of variability.
- Branches appearing in bifurcation diagrams correspond to different solutions including a high response period (subharmonic solutions). They are characterized by the largest amplitudes of the displacement of a flexible cantilever beam but they do not always turn out to be the most effective solution from the point of view of harvesting energy. On the other hand, their presence is beneficial for the broadband energy harvesting effect. Additionally, the phenomenon of transient chaos was identified.
- With the increase in the value of the parameter *β*, a reduction in the ability to harvest energy from vibrating mechanical devices is observed. However, this statement refers to the maximum values recorded on the piezoelectric electrodes.
- Taking into account the narrowband indicators characterizing the efficiency of energy harvesting (Figure 17), the most favorable configuration of the elastic elements of the energy harvesting system with a star-shaped structure of elastic elements with variable configuration should be determined by the parameter *β* = 2.

Systems in which the potential barrier is shaped by means of elastic elements may show sensitivity to the operation of articulated joints. For this reason, these critical structural nodes require precise execution, which, in consequence, may significantly increase the costs of making such an energy harvesting system. However, the undoubted advantage is the possibility of almost any shape of the potential function. Energy harvesting systems composed of variable springs require proper maintenance. Depending on the environment in which they will operate, they can be installed in waterproof, dustproof, intrinsically safe enclosures. As a result, the influence of the external environment can be practically eliminated to zero. The use of an appropriate casing enables the installation of the system and its maintenance-free operation in water and dusty and dangerous environments—e.g., methane mines, etc.

The work is in progress with an experimental program in order to validate the theoretical approach here pursued with a metrological characterization.

## Figures and Tables

**Table 1:** Geometric and physical parameters of the model.

Name	Symbol	Value
Inertial element (mass) loading the beam	*m*	0.005 kg
Energy losses in a mechanical system	δ	0.0676 Nsm^−1^
Stiffness of the compensation springs	*c*_1_	70 Nm^−1^
Stiffness ratio of elastic elements	*μ*_1_	1.2
	*μ*_2_	0.5
	*μ*_3_	0.1
Design parameter	*a*_0_	0.015 m
Equivalent resistance of the piezoelectric converter	*R_Z_*	1.1 × 10^6^ Ω
Equivalent capacity of the piezoelectric converter	*C_P_*	72 nF
Electromechanical constant of piezoelectric converter	*k_P_*	3.985 × 10^−5^ N/V

## Data Availability

Data are contained within the article.
